# Differential Diagnosis of Cesarean Scar Pregnancies and Other Pregnancies Implanted in the Lower Uterus by Ultrasound Parameters

**DOI:** 10.1155/2020/8904507

**Published:** 2020-11-24

**Authors:** Kangning Li, Qing Dai

**Affiliations:** ^1^Department of Medical Ultrasound, Tongji Hospital, Tongji Medical College, Huazhong University of Science and Technology, Wuhan, Hubei Province, China 430030; ^2^Department of Medical Ultrasound, Peking Union Medical College Hospital, Chinese Academy of Medical Science & Peking Union Medical College, Beijing, China 100730

## Abstract

**Purpose:**

Cesarean scar pregnancy is an extremely rare type of ectopic pregnancy implanted in the myometrium at the site of a previous cesarean section scar. On the other hand, pregnancies are considered low implantations if they are identified in the lower third of the uterus without the sac implanted into the scar and have a better prognosis. Early diagnosis of both types of pregnancies can help avoid serious complications. This study is aimed at investigating the significance of transvaginal ultrasound in the differential diagnosis of cesarean scar pregnancies and pregnancies implanted in the lower uterus.

**Methods:**

Ninety-three patients with an average age of 32.7 years (range, 24–43 years) were enrolled in this study, including 66 cesarean scar pregnancies and 27 other pregnancies implanted in the lower uterus, and they were examined by transvaginal ultrasound.

**Results:**

We observed significant differences in the relationship between the cesarean sac and the scar, the source of the trophoblastic blood flow, and the thickness of the residual muscle between the cesarean scar pregnancy group and the lower uterus pregnancy group. We established the logistic model to improve the differential diagnosis of cesarean scar pregnancies and pregnancies implanted in the lower uterus.

**Conclusions:**

Transvaginal ultrasound is recommended in early pregnancy, especially for patients who have undergone a previous cesarean section delivery.

## 1. Introduction

Cesarean scar pregnancy is one type of ectopic pregnancy in which the gestational sac is implanted into the prior cesarean scar, and its morbidity has been rapidly rising with the increased rate of cesarean deliveries [1, 2]. Cesarean scar pregnancy with positive embryonic/fetal heart activity managed expectantly is associated with a high burden of maternal morbidity [3]. Women with a prior cesarean scar pregnancy have a high risk of recurrence, miscarriage, preterm birth, and placenta accreta spectrum, but it remains unclear whether different types of management impact reproductive outcome [4]. Therefore, it is very important to manage cesarean scar pregnancy properly.

Ultrasound is regarded as the first-line of examination for cesarean scar pregnancies. The following diagnostic criteria proposed by Godin et al. are most widely accepted: (i) an empty uterine cavity, without contact with the sac; (ii) a clearly visible empty cervical canal, without contact with the sac; (iii) the presence of the sac in the anterior uterine isthmus; and (iv) an absence of or a defect in the myometrial tissue between the bladder and the sac [5]. Later, Vial et al. added a special case, namely, gestational sacs that grow into the uterine cavity, rather than into the scar, which had a relatively better prognosis [6]. However, it is still difficult to differentiate between cesarean scar pregnancies and pregnancies implanted in the lower uterus, which includes the implantation of gestational sacs in the lower anterior uterus close to the scar or the lower posterior uterus. This is a great challenge in the clinic, because whether the sac is truly implanted into the scar is directly related to the prognosis. Therefore, this study is aimed at establishing a new diagnostic model to differentiate between these two similar situations by ultrasound imaging.

## 2. Subjects and Methods

### 2.1. Subjects

Women that underwent routine early pregnancy (from 5 w + 0 d to 9 w + 6 d) transvaginal ultrasound examination from January 2011 to May 2018 were prospectively collected. The inclusion criteria were as follows: (i) a history of one or more cesarean deliveries; (ii) the gestational sac was implanted in the lower uterus, as assessed by ultrasound. The exclusion criteria were as follows: (i) a cervical pregnancy, an incomplete abortion, or gestational trophoblastic disease that could not be ruled out; (ii) a uterine artery embolization or medical treatment that was carried out before dilatation and curettage. This study was approved by the Ethics Committee and conducted in accordance with the Declaration of Helsinki and its revisions. Written informed consent was obtained from all the patients.

### 2.2. Procedures

**Figure 1:**
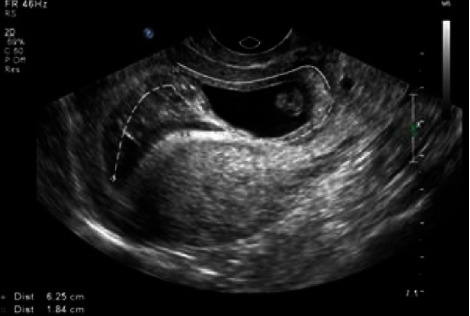
Ultrasound image of the implantation site. A small amount of fluid was observed inside the endometrial cavity, which delineated the border of the hyperechoic ring around the conceptus. The dotted line indicated the endometrial lumen, and the solid line indicated the implantation site.

Philips iU22 color Doppler ultrasound instrument with a C9-4 transducer was used. First, we identified the implantation site, which could be divided into five types: the fundus, the anterior part, the posterior part, the lower anterior part, and the lower posterior part. The first three types of implantation sites were excluded from our study, and the implantation site was examined as described previously [7]. In the sagittal view of the uterus, the implantation site manifested as a hyperechogenicity ring that occupied one side of the gestational sac, opposite to the displacement direction of the uterine cavity (Figure 1), which was considered as the maternal decidual reaction, the start site of maternal-fetal circulation, and the original site of placental formation and development.

Second, we assessed the relationship between the gestational sac and the cesarean scar, which could be divided into four types: away from the scar, close to the scar, across the scar, and into the scar. The first type of scar was excluded from our study.

**Figure 2:**
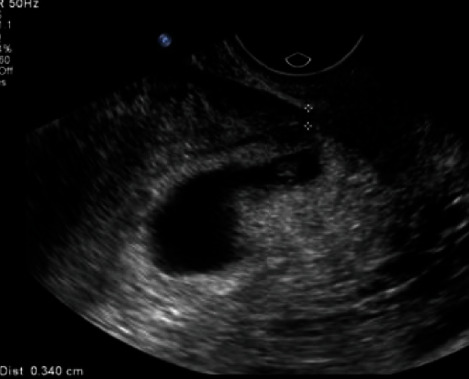
Measurement of the thickness of the remaining myometrium over the scar.

Third, we measured the thickness of the residual muscle above the scar, which was the shortest distance between the uterine serosa and the chorionic villi [8]. In brief, we positioned the cursors on the inner side of the uterine serosa (hyperechogenicity) and the outer side of the chorionic villi (hyperechogenicity) and measured the area of hypoechogenicity, which corresponded to the residual muscle (Figure 2).

**Figure 3:**
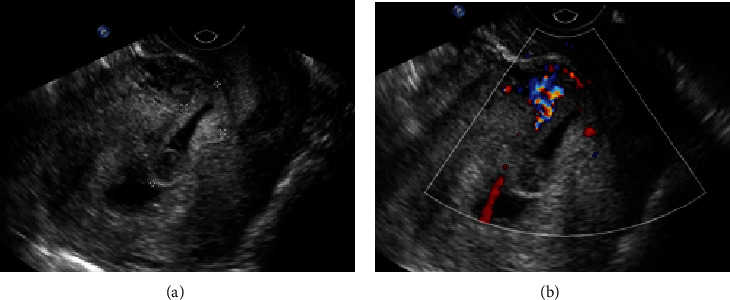
A 28-year-old female (gravida 4, para 2) presented with amenorrhea for 42 days. (a) The sagittal grayscale image showed that the sac was implanted in the lower endometrial cavity, protruding into the scar. There was a small amount of fluid in the uterine fundus. (b) The color Doppler image showed the trophoblastic blood flow from the lower anterior uterus. Chorionic villi were observed within the scar with a laparoscopy combined with uterine artery embolization.

**Figure 4:**
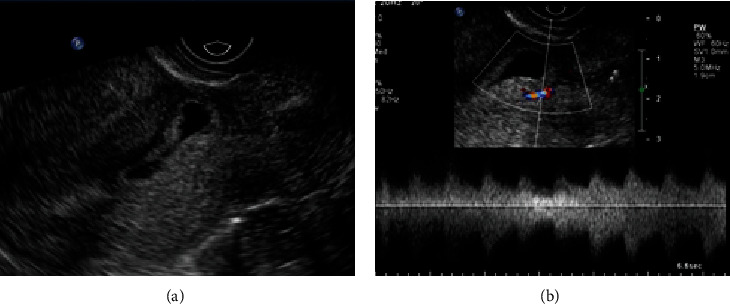
A 36-year-old female (gravida 4, para 1) presented with amenorrhea for 47 days. (a) The sagittal grayscale image showed that the sac was implanted in the lower endometrial cavity, protruding into the scar. (b) The color Doppler image showed the trophoblastic blood flow from the lower posterior uterus. Chorionic villi were not visible inside the scar with a laparoscopy.

**Figure 5:**
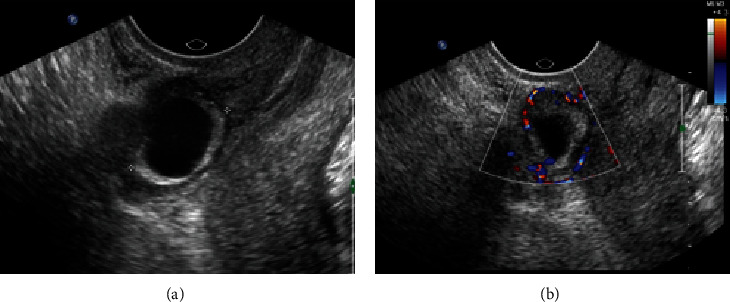
A 29-year-old female (gravida 2, para 1) presented with amenorrhea for 44 days. (a) The sagittal grayscale image showed that the sac was implanted in the lower endometrial cavity, protruding into the scar. A small amount of fluid was seen around the posterior of the conceptus, suggesting that the sac was implanted into the scar. (b) The color Doppler image showed the trophoblastic blood flow surrounding the sac, which made it difficult to estimate the source of the trophoblastic blood. Chorionic villi were visible within the scar with a laparoscopy combined with uterine artery embolization.

Fourth, we identified the source of the trophoblastic blood flow, which could be divided into three sources: from the lower anterior lower uterus (Figure 3), the lower posterior uterus (Figure 4), and unknown (Figure 5). When the trophoblastic blood flow could not be differentiated from the myometrial blood flow by the color Doppler mode, the pulse Doppler function was used to provide additional information. Typical trophoblastic blood flow usually showed a high-velocity (peak velocity > 20 cm/s) and a low-resistance (pulsatility index ≤ 1) frequency spectrum, consistent with normal pregnancies [9]. If it was difficult to identify the source of the trophoblastic blood flow by both the color and pulse Doppler ultrasound, it was defined as unknown.

All the examinations were performed by an experienced ultrasonographer, and all the clinical information was blind to the examiner. The subsequent treatment outcomes were followed-up. The pathological diagnostic standard was that the chorionic villi were confirmed to be within the cesarean scar, as observed with a laparoscopy. The clinical diagnostic standard was that the volume of lost blood was greater than the upper limit of normal (100 ml) during dilatation and curettage, usually combined with debris. If a case did not meet these criteria, it was considered as a pregnancy implanted in the lower uterus, which was subdivided into pregnancies with the gestational sac implanted in the lower anterior uterus close to the scar and pregnancies with the gestational sac implanted in the lower posterior uterus.

### 2.3. Statistical Analysis

All statistical analyses were performed using IBM SPSS 22.0 software. The measurement data were expressed as means ± standard deviation and compared by *t*-test between the cesarean scar pregnancies and the pregnancies implanted in the lower uterus. The categorical data were presented as frequencies and compared by the chi-square test between the two groups. The sensitivity, specificity, positive predictive value, negative predictive value, and accuracy of the ultrasound indicators were calculated. Logistic regression analysis was used to establish a diagnostic model, and the diagnostic efficiency of the model was evaluated using the receiver operating characteristic curve. *P* values < 0.05 were considered significant.

## 3. Results

Ninety-three patients with an average age of 32.7 years (range, 24–43 years) were enrolled in our study, including 66 cesarean scar pregnancies and 27 other pregnancies implanted in the lower uterus. Compared to females with pregnancies implanted in the lower uterus, more females with cesarean scar pregnancies chose laparoscopy as their treatment method (*P* < 0.05). There were no significant differences in the age, the gestational age, the number of cesarean deliveries, and vaginal bleeding between the two groups (Table 1).

We observed significant differences in the relationship between the cesarean sac and the scar, the source of the trophoblastic blood flow, and the thickness of the residual muscle between the cesarean scar pregnancy group and the lower uterus pregnancy group. There was no significant difference in the implantation site between the two groups (Table 2).

**Figure 6:**
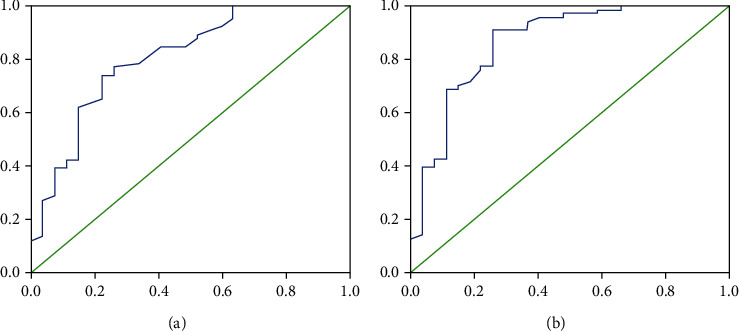
Analysis of the receiver operating characteristics of the thickness of the residual muscle (a) and the logistic regression model (b).

When the thickness of the residual muscle was regarded as the independent diagnostic indicator, the area under the receiver operating characteristic (ROC) curve was 0.806, which was taken as a moderate diagnostic value (Figure 6(a)). When 2.35 mm was considered as the cut-off value, the sensitivity, specificity, positive predictive value, negative predictive value, and accuracy were 74.2%, 77.8%, 89.1%, 55.3%, and 75.3%, respectively.

The relationship between the gestational sac and the scar, the source of trophoblastic blood flow, and the thickness of the residual muscle were selected as the diagnostic indicators using the logistic regression model (Table 3).

The area under the ROC curve of the logistic regression model was 0.863, which was higher than that of the thickness of the residual muscle as an independent indicator (Figure 6(b)).

When *P* = 0.680 was considered as the cut-off value, the diagnostic accuracy was 86%. The sensitivity, specificity, positive predictive value, and negative predictive value were 90.9%, 74.1%, 89.6%, and 76.9%, respectively, which were higher than those of each independent indicator (Table 4).

## 4. Discussion

The principle of treatment for females with cesarean scar pregnancies is to terminate the pregnancy in order to avoid serious complications such as uterine rupture and massive hemorrhage [10]. Other pregnancies implanted in the lower uterus may develop into placenta previa or placenta accrete. However, follow-up is acceptable with close monitoring and reasonable assessment of the risks for those determined to keep the babies [11]. Therefore, it is crucial to make differential diagnosis between a cesarean scar pregnancy and a pregnancy implanted into the lower uterus, as their outcomes are not completely the same.

Most studies have used the relationship between the gestational sac and the cesarean scar to diagnose cesarean scar pregnancies. If the gap between the sac and the scar vanishes, this is taken to mean that the sac is implanted into the scar [12]. This indicator can effectively differentiate pregnancies implanted in the lower anterior uterus near the scar from cesarean scar pregnancies. However, when the gestational sac is implanted in the lower posterior uterus, this can create the false-positive disappearance of the gap, which makes this indicator highly sensitive and unspecific.

The thinning myometrium above the cesarean scar is another crucial indicator of myometrial invasion and implantation into the scar. A case series has reported that two-thirds of cesarean scar pregnancies have a thinning myometrium less than 5 mm in thickness [13]. In our study, when the remaining myometrial depth of 2.35 mm was regarded as the diagnostic indicator, the area under the curve of ROC was 0.806, which was taken as a moderate diagnostic value. However, when the cesarean scar heals poorly, a myometrial defect may develop, which can also lead to a thinning myometrium. Interestingly, Osser et al. reported that the probability of the depth of the remaining myometrium less than 2.2 mm is 14%, 23%, and 43% in a first, second, or third cesarean delivery, respectively [8, 14]. Therefore, the thickness of residual muscle is not a good indicator.

Since all the above indicators represent indirect signs, if we can directly observe the implantation site, it may help us to correctly diagnose the cesarean scar pregnancy. Abdallah et al. proposed that the implantation site can manifest as a hyperechogenicity ring that occupies one side of the gestational sac, opposite to the displacement direction of the uterine cavity [7]. According to our observations, with the growing of the gestation sac and the decidua capsularis getting gradually closer to the decidua parietalis, the structure of the uterine cavity disappeared. When there is no fluid in the uterine cavity, it is difficult to detect the displacement direction of the uterine cavity line and thus the hyperechogenicity ring represented as the implantation site. This may be the reason behind the finding that there was no significant difference in the subjective judgment of the implantation site between the two groups. It was proposed that transvaginal ultrasound combined with color and pulsed Doppler assessment in early gestation provided better opportunity of detecting cesarean scar pregnancies [15].

Currently, there is sufficient evidence to demonstrate that cesarean scar pregnancies and placental implantations share a common histology, and they are considered by many investigators to actually be the same condition occurring at different periods [16]. During the second trimester, the sinus full of turbulent blood flow in the placenta near the myometrium and the interruption of bladder uterine serous above the placenta with increasing blood flow are two reliable diagnostic indicators for placenta accrete [17, 18]. The aforementioned two signs can also be observed during the first trimester [19]. This can be explained by the fact that the implantation site is also the original site of placental formation and development. Other signs suggesting cesarean scar pregnancies include negative sliding organ sign, i.e., the absence of gestational sac mobility upon gentle pressure with a probe of transvaginal ultrasound in the vagina. These signs facilitate the exclusion of other diagnoses such as cervical-isthmic pregnancy or inevitable miscarriage [20]. Accordingly, our study attempted to observe the peripheral trophoblastic blood flow of the gestational sac to identify the implantation site and its relationship with the cesarean scar. Therefore, the source of the trophoblastic blood flow can effectively differentiate cesarean scar pregnancies from pregnancies implanted into the lower posterior uterus. However, it is still difficult to identify pregnancies implanted into the lower anterior uterus close to the scar.

Regardless of the single diagnostic indicator, there are several limitations in differentiating between cesarean scar pregnancies and other pregnancies implanted into the lower uterus. As a result, this study established the logistic model in order to increase the diagnostic efficiency. The area under the ROC curve of the logistic model was 0.863, which was higher than 0.806, the area under the ROC curve of the thickness of the remaining myometrium as an independent diagnostic indicator. The sensitivity, specificity, positive predictive value, and negative predictive value of the logistic model were higher than those of each independent diagnostic indicator. Therefore, the logistic model developed in this study can better distinguish cesarean scar pregnancies from other pregnancies implanted into the lower uterus, avoiding the incorrect diagnosis of cesarean scar pregnancies and unnecessary uterine artery embolism or termination of the pregnancy.

There are several limitations in this study. With increased clinical experience, gynecologists often prefer to choose a less invasive treatment such as ultrasound-guided curettage rather than laparoscopy. Therefore, several patients did not receive pathological results and a confirmation of the actual implantation site. Jurkovic et al. systematically reviewed the volume of lost blood from 191 females with cesarean scar pregnancies who undertook dilation and curettage guided by ultrasonography. The median volume of lost blood was 100 ml, which is significantly more than the amount of lost blood during general dilation and curettage [21]. This can be explained by the fact that the chorionic villi are implanted into the myometrium, and their separation from the implantation site can easily cause bleeding. Furthermore, the myometrium above the scar is weakly contractile, which can aggravate bleeding. Consequently, it is critical to consider the amount of lost blood during dilation and curettage as the alternative diagnostic standard when the gestational sac is located in the lower uterus. Although several implantation sites of the cesarean scar pregnancy are superficial, in this situation, the amount of lost blood can be very low, and the cesarean scar pregnancy can be mistaken for a pregnancy implanted in the lower uterus. Thus, the model can truly reflect the prognosis and not affect clinical decision.

In conclusion, combined use of ultrasound indicators, such as the relationship between the gestational sac and the cesarean scar, the source of the trophoblastic blood flow, and the thickness of the residual muscle, could improve differential diagnosis between cesarean scar pregnancies and other pregnancies implanted in the lower uterus.

## Figures and Tables

**Table 1:** Characteristics of patients.

	Cesarean scar pregnancies	Other pregnancies implanted in the lower uterus	*P* value
Age (y)	33.2 ± 6.2	34.4 ± 5.6	0.408
Gestational age (d)	46.5 ± 6.2	49.0 ± 7.3	0.106
No. of cesarean delivery			
1	56	23	0.967
2	10	4	
Vaginal bleeding (%)	37.9%	51.9%	0.215
Treatment method			<0.001
Dilation and curettage	17	19	
Laparoscopy	49	8	

**Table 2:** Comparison of sonographic data between the groups.

	Cesarean scar pregnancies	Other pregnancies implanted in the lower uterus	*P* value
Implantation site			0.316
Lower anterior part	15	3	
Lower posterior part	7	5	
Unknown	44	19	
Gestational sac in relation to the scar			<0.0001
Close to or across the scar	4	11	
Inside the scar	62	16	
Trophoblastic blood flow			<0.0001
Lower anterior part	58	11	
Lower posterior part	3	12	
Unknown	5	4	
Thickness of residual muscle (mm)	1.6 ± 1.0	3.4 ± 1.8	<0.0001

**Table 3:** Results of the logistic regression model.

Risk factor	Regression coefficient	Standard error	Wald chi-square value	*P* value	OR
Gestational sac in relation to the scar	1.599	0.827	3.739	0.053	4.946
Trophoblastic blood flow			6.051	0.049	
Type I^a^	1.846	1.126	2.685	0.101	6.332
Type II^b^	2.048	0.837	5.993	0.014	7.755
Thickness of the residual muscle	-0.755	0.309	5.967	0.015	0.470
Constant	-1.900	1.992	0.909	0.340	0.150

^a^Comparison between the trophoblastic blood from the lower posterior uterus and unknown; ^b^comparison between the trophoblastic blood from the lower posterior uterus and the lower anterior uterus.

**Table 4:** The diagnostic accuracy of independent indicators and the logistic regression model.

	Sensitivity	Specificity	Positive predictive value	Negative predictive value	Accuracy
Implantation site	93.9%	40.7%	79.5%	73.3%	78.5%
Sac in relation to the scar	87.9%	44.4%	84.1%	80.0%	75.3%
Thickness of residual muscle	74.2%	77.8%	89.1%	55.3%	75.3%
Logistic regression model	86.3%	90.9%	74.1%	89.6%	76.9%

## Data Availability

All data are available upon request.
